# The Calcium-Dependent Protein Kinase TaCDPK7 Positively Regulates Wheat Resistance to *Puccinia striiformis* f. sp. *tritici*

**DOI:** 10.3390/ijms25021048

**Published:** 2024-01-15

**Authors:** Farhan Goher, Xingxuan Bai, Shuai Liu, Lefan Pu, Jiaojiao Xi, Jiaqi Lei, Zhensheng Kang, Qiaojun Jin, Jun Guo

**Affiliations:** 1 State Key Laboratory of Crop Stress Biology for Arid Areas, College of Plant Protection, Northwest A&F University, Yangling 712100, China; goherfarhan@nwafu.edu.cn (F.G.); baixingxuan@nwafu.edu.cn (X.B.); liushuai0203@nwafu.edu.cn (S.L.); lefanp@nwafu.edu.cn (L.P.); jiaojiaoxi@nwafu.edu.cn (J.X.); ljq15207144758@nwafu.edu.cn (J.L.); kangzs@nwsuaf.edu.cn (Z.K.); 2 Key Laboratory of Plant Protection Resources and Pest Management of Ministry of Education, College of Plant Protection, Northwest A&F University, Yangling 712100, China

**Keywords:** biotic stress, wheat, stripe rust, *TaCDPK7*, resistance

## Abstract

Ca^2+^ plays a crucial role as a secondary messenger in plant development and response to abiotic/biotic stressors. Calcium-dependent protein kinases (CDPKs/CPKs) are essential Ca^2+^ sensors that can convert Ca^2+^ signals into downstream phosphorylation signals. However, there is limited research on the function of CDPKs in the context of wheat–*Puccinia striiformis* f. sp. *tritici* (*Pst*) interaction. In this study, we aimed to address this gap by identifying putative *CDPK* genes from the wheat reference genome and organizing them into four phylogenetic clusters (I-IV). To investigate the expression patterns of the *TaCDPK* family during the wheat–*Pst* interaction, we analyzed time series RNA-seq data and further validated the results through qRT-PCR assays. Among the *TaCDPK* genes, *TaCDPK7* exhibited a significant induction during the wheat–*Pst* interaction, suggesting that it has a potential role in wheat resistance to *Pst*. To gain further insights into the function of *TaCDPK7*, we employed virus-induced gene silencing (VIGS) to knock down its expression which resulted in impaired wheat resistance to *Pst*, accompanied by decreased accumulation of hydrogen peroxide (H_2_O_2_), increased fungal biomass ratio, reduced expression of defense-related genes, and enhanced pathogen hyphal growth. These findings collectively suggest that *TaCDPK7* plays an important role in wheat resistance to *Pst*. In summary, this study expands our understanding of wheat CDPKs and provides novel insights into their involvement in the wheat–*Pst* interaction.

## 1. Introduction

Calcium ions (Ca^2+^) are important cellular ionic species that function as a second messenger in signaling transduction pathways when plants are encountered with a variety of developmental and environmental stimuli [1]. In response to these stimuli, the cytosolic free Ca^2+^ levels rise drastically [2], and these changes are sensed by Ca^2+^ sensors. The Ca^2+^ sensor proteins are divided into two categories: those that relay calcium signals, such as calcineurin B-like/CBL-interacting protein kinases (CBL/CIPKs) and calmodulin proteins (CaMs), while the others function as sensor protein kinases, including calcium-dependent protein kinases (CDPKs/CPKs) and calmodulin-dependent protein kinases (CaMKs) [3]. In contrast to non-catalytic relay sensors like CaMs and CBLs, which transduce calcium signals to target proteins, CDPKs have the unique ability to directly communicate Ca^2+^ signals. They achieve this by binding calcium to the EF hands located at their C-terminus and phosphorylating substrates through the catalytic kinase domain at their N-terminus. Typical CDPKs have an N-terminal domain followed by a protein kinase domain, an auto-inhibitory domain, and a C-terminal regulatory calmodulin-like domain that contains one to four EF-hand motifs responsible for binding Ca^2+^ [4]. Additionally, CDPKs possess myristylation and palmitoylation sites at the beginning of their N-terminal domains, which help them attach to or detach from membranes. The N-terminal domains of CDPKs are highly variable in length and amino acid content, contributing to their diverse functional roles [5]. The protein kinase domain, located immediately upstream of the auto-inhibitory domain, comprises a catalytic Ser/Thr protein kinase domain with an ATP binding site. In the absence of Ca^2+^, the auto-inhibitory domain acts as a pseudo substrate of the kinase domain and interacts with its active sites to keep CDPKs inactive [6]. Upon Ca^2+^ binding, CDPKs undergo a conformational change that disrupts the inhibitory interaction between the auto-inhibitory domain and the active site, leading to the activation of the kinase domain. In addition to their significant roles in developmental processes such as pollen tube formation, root expansion, stem elongation, and cell division/differentiation, CDPKs and related kinases (CRKs) are also actively involved in responding to biotic/abiotic stresses and phytohormone-mediated signaling pathways [7,8]. For instance, the AtCPK10 protein participates in Ca^2+^- and ABA-mediated stomatal movements during drought stress in Arabidopsis [9], while AtCPK1 is associated with the accumulation and constitutive biosynthesis of salicylic acid (SA) and regulates defense through modulation of disease resistance gene expression [10].

CDPKs play a crucial role in plant defense responses against pathogens, encompassing oxidative burst, alterations in phytohormone synthesis and signaling, and modulation of gene expression [11]. They exhibit both positive and negative regulatory effects on plant defense mechanisms [12]. In wheat, *TaCPK2-A* is required for resistance to powdery mildew, while its overexpression in rice enhances resistance to bacterial blight (caused by *Xanthomonas oryzae* pv. *oryzae*, *Xoo*) by modulating the expression of the transcription factor WRKY45-1 [13]. In a recent study, silencing of *TaCDPK27* improves wheat resistance to powdery mildew infection [14]. Overexpression of *AtCPK1* in *Arabidopsis* confers broad-spectrum defense against fungi and bacteria [10]. Moreover, overexpression of *OsCPK4* in rice enhances resistance to blast disease caused by *Magnaporthe oryzae* [15]. The *CDPK2* and *CDPK3* in Nicotiana species have been shown to play essential roles in the initiation of programmed cell death (PCD) triggered by the Avr4 or Avr9 elicitors, which are specific to particular races of *Cladosporium fulvum* [16]. Interestingly, the stable expression of *StCPK5-VK* in transgenic potatoes resulted in enhanced resistance against the hemi-biotrophic oomycete *Phytophthora infestans*, while it led to reduced resistance against the necrotrophic fungus *Alternaria solani* [17]. Recent research has identified *Arabidopsis* CPK3 as a key regulator in both pathogen-associated molecular patterns (PAMPs)-triggered immunity (PTI) and effectors-triggered immunity (ETI) [18]. *Arabidopsis* CPK4, 5, 6, and 11 collectively and redundantly contribute to PAMP-induced resistance against *Pseudomonas syringae* [19]. On the other hand, CDPKs can also exhibit negative regulatory effects during pathogen interactions. For instance, overexpression of the *OsCPK12* in rice enhances sensitivity to both avirulent and virulent strains of *M. oryzae* [7], while overexpression of an auto-active variant of *HvCDPK3* in barley leads to increased susceptibility to powdery mildew [20].

CDPKs are plant-specific proteins that have been widely identified from algae to flowering plants [4]. Despite extensive studies on CDPKs in other plants, our understanding about CDPKs in bread wheat, the third-largest cultivated crop in the world remains limited [21,22]. Earlier, twenty wheat CDPK genes were identified when the whole genome sequence of wheat was not available [22]. However, after the complete sequencing of the hexaploidy wheat genome in 2018 [23], a comprehensive investigation of CDPKs in wheat was needed.

Stripe rust, caused by the fungus *Puccinia striiformis* f. sp. *tritici* (*Pst*), is the most devastating fungal disease of wheat, leading to yield losses of up to 50% [24]. The control of stripe rust in wheat involves various strategies, including chemical and biological methods. The most cost-effective and environmentally friendly approach is to utilize resistant genes. However, the rapid evolution of *Pst*, and emergence of new races can breakdown the resistance provided by race-specific resistant gene(s) in wheat. For example, recently emerged *Pst* races, TSA-6 [25], and TRVR20-5 [26], have shown virulence to the wheat *Yr5* gene, which has been known to convey resistance to *Pst* races worldwide and has been utilized in the international wheat breeding programs focused on strip rust resistance. Consequently, it is of utmost importance to investigate and discover novel genes that exhibit resistance to *Pst*. Notably, no *TaCDPK* genes have been reported to be involved in wheat resistance against stripe rust so far. Therefore, there is a need to investigate the functional roles of wheat *CDPKs* in combating this notorious pathogen.

In this study, we conducted a comprehensive genome-wide analysis of *CDPK* genes in the bread wheat reference genome, resulting in the identification of 79 putative wheat *CDPK* genes, which were categorized into four clusters based on phylogenetic analysis. We examined the synteny, analyzed gene structure, motif organization, and exon–intron composition. Expression analyses revealed that *Pst* infection regulated the expression of several of these 79 wheat genes. Furthermore, functional characterization using VIGS indicated a positive role of *TaCDPK7* in the wheat–*Pst* interaction, possibly by inhibiting the expression of ROS-scavenging genes while promoting the expression of PR genes. Our study lays the basis for further characterization of the function of CDPKs in wheat during stress responses and enhances our understanding of the role of CDPKs in plant defense against pathogens.

## 2. Results

### 2.1. Mining of CDPK Members from Wheat Genome

To identify wheat CDPKs, we used the *Arabidopsis* and rice CDPK protein sequences as queries to search against the wheat genome database at http://plants.ensembl.org/ (accessed on 6 September 2022) using the Local BLASTP program available. After eliminating sequence redundancies, we obtained a total of 79 putative TaCDPKs that contain the corresponding signature domain (SMART acc. No. SM000220 and InterPro acc. No. IPR000719). The length of the coding sequences (CDS) of *TaCDPKs* range from 1384 bp to 1885 bp.

Since rice is considered a close relative of wheat in the classification system, the 79 identified genes were named in ascending order according to their phylogenetic relationships with the rice CDPK family [27] (Table S1). All the identified *TaCDPK*s are located on the wheat chromosomes 1 to 7, except for *TaCDPK4UN*, which is located at an unknown locus. Among them, 24 genes are located on the 5th chromosome, followed by 17 genes on the 2nd chromosome, 11 genes on the 4th chromosome, 9 genes each on the 1st and 3rd chromosomes, 5 genes on the 6th chromosome, and 3 genes on the 7th chromosome. Therefore, chromosome 5 has the maximum number of *TaCDPKs*.

### 2.2. Phylogeny and Computational Characterization of Wheat CDPKs

**Figure 1:**
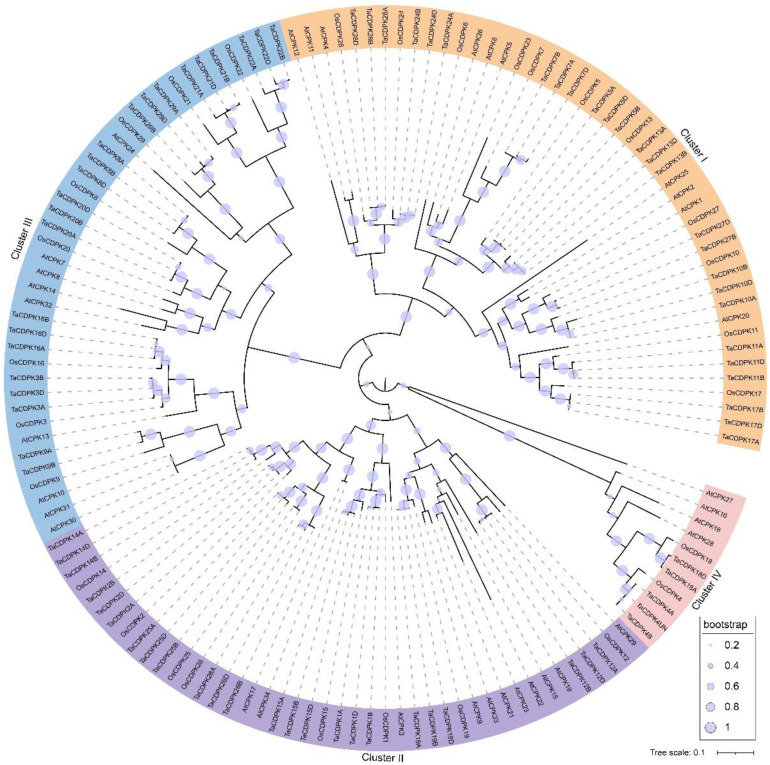
Phylogenetic relationship of TaCDPK (calcium-dependent protein kinase), OsCDPK, and AtCDPK proteins. Maximum likelihood, mid-rooted tree was constructed using MEGA 6, with 0.1 scale bars. Distinct colors indicate different clusters (I–IV).

To determine the evolutionary relationship of CDPKs in wheat, rice, and *Arabidopsis*, a phylogenetic tree was constructed using full-length protein sequences. Similar to rice and *Arabidopsis*, the wheat CDPKs were grouped into four clusters in the phylogenetic tree. Among these clusters, cluster I was the largest, containing 47 members, with 26 from wheat, 11 from rice, and 10 from *Arabidopsis* (Figure 1). Second largest is cluster II having 43 members in total, and cluster III contained 42 members. Cluster IV is the smallest one, comprising 10 members, 5 from wheat, 2 from rice, and 3 from *Arabidopsis*. Notably, all the wheat *CDPK* genes have complete triplet copies of their sub-genomes (BB, AA, DD) except for two genes, *TaCDPK18* from cluster IV and *TaCDPK27* from cluster I, which were found to lack their B and A sub-genome homologous triplets’ copies, respectively. The phylogenetic analysis revealed that the wheat CDPK genes displayed closer relationships with the CDPK genes in rice (monocot) compared to those in *Arabidopsis* (dicot). In light of this, the naming of TaCDPK proteins was assigned based on their homologies with rice CDPKs rather than *Arabidopsis*. Furthermore, we investigated the putative orthologous similarity index between wheat and rice CDPKs based on the phylogenetic tree. The highest similarity index, 95%, was observed between *TaCDPK16D* and *OsCDPK16*, while the lowest index, 65%, was found between *TaCDPK22D* and *OsCDPK22*. Notably, nineteen members of the *TaCDPK* family exhibited a similarity index above 90% (Table S2).

To examine the gene structures of *TaCDPK*s, we analyzed the exon–intron structures by comparing their cDNA sequences with the corresponding genomic sequences. The number of exons in *TaCDPK*s ranged from three to twelve per gene, while the introns varied from two to eleven per gene (Figure S1). We used the TBtools kit to identify gene duplication events (Figure S2). Tandem duplication is the likely cause of having two or more homologous genes on the same chromosome within a 200 kb region [28]. Since chromosome 5 harbors the maximum number of *TaCDPK*s, it is likely that tandem duplications have occurred on this chromosome. Four possible tandem duplication events were identified for eight *TaCDPK* genes on chromosome 5 (Table S5), and five out of these eight genes belonged to cluster I. Furthermore, a considerable number of *TaCDPK*s seemed to have arisen from segmental duplication events (Figure S2), suggesting that both tandem and segmental duplications played a role in the expansion of the wheat CDPK family. In conclusion, our findings indicate that a significant number of *TaCDPK* genes may have undergone duplication events during evolution, shedding light on the evolutionary and functional potential of *TaCDPK* genes.

To gain further insights into the expression regulation patterns of wheat *CDPK* genes, we conducted in silico investigations of the possible *cis*-elements present in the promoters of each *TaCDPK* family member. The analysis revealed a diverse range of *cis*-elements in the promoters of *TaCDPK*s, including plant hormone stimulus-responsive elements (such as P-box, TGACG motif, ABRE, TCA element, CGTCA motif, and GARE motif) and biotic/abiotic stress response elements (such as MBS, TC-rich repeats, Circadian, and LTR) (Figure S3). Additionally, based on the predicted functions of all *TaCDPK*s, the *cis*-elements were categorized into major groups, comprising hormone-responsive *cis*-elements, followed by stress-responsive *cis*-elements, with other categories represented to a lesser extent (Figure S4). The identification of a substantial number of *cis*-elements associated with hormone and stress responsiveness highlights the signaling role of the wheat CDPK gene family in response to hormonal stimuli and abiotic/biotic stress conditions.

Domains and motifs play crucial roles in protein interactions, transcriptional activity, and DNA binding for transcription factors [29]. In our study, we utilized the MEME suite to identify conserved motifs within the TaCDPK proteins. Among the identified motifs, motifs 1 and 2 corresponded to the protein kinase domain (IPR000719) were highly conserved across all members of the TaCDPK family (Figure S5). Additionally, motifs 5, 7, and 9, which corresponded to the EF-hand domain (IPR002048) and EF-hand domain pair (IPR011992), were also observed throughout the entire family. These conserved motifs provide further evidence of the functional importance of protein kinase and EF-hand domains in the TaCDPK family.

### 2.3. Expression Patterns of TaCDPKs during Wheat–Pst Interaction

**Figure 2:**
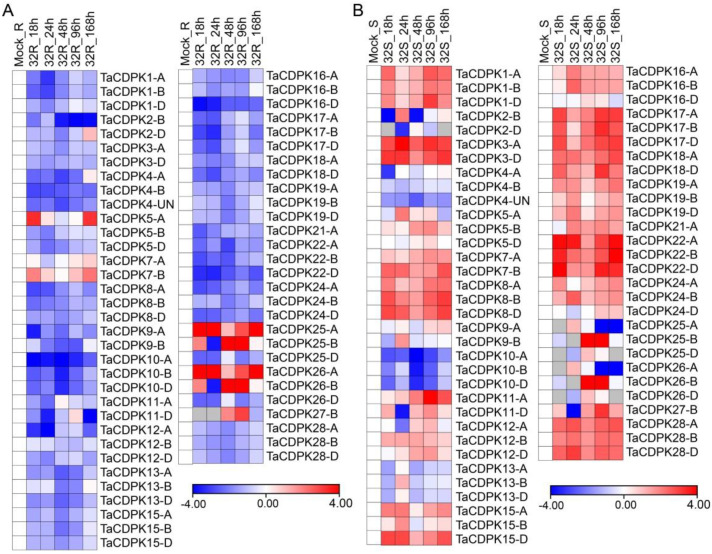
Transcriptional levels of *TaCDPK*s in compatible and incompatible interactions between wheat and *Puccinia striiformis* f. sp. *tritici* (*Pst*). The expression patterns of *TaCDPK*s analyzed using logFC [log_2_^(foldchange)^] at 0, 18, 24, 48, 96, and 168 hpi in compatible and incompatible interactions in time series dual RNA-seq data. (**A**) The incompatible combination 32R, (**B**) the compatible combination 32S. Downregulation and upregulation are indicated with blue and red colors, respectively. The white color shows expression patterns that are comparable to those after mock treatment.

To investigate the expression patterns of *TaCDPK*s during the wheat–*Pst* interaction, we analyzed their transcript levels using RNA-seq data obtained from wheat samples at 0, 18, 24, 48, 96, and 168 hpi (hours post inoculation). *TaCDPK2A*, *TaCDPK3B*, *TaCDPK7D*, *TaCDPK9D*, *TaCDPK11B*, *TaCDPK14A*,*B*,*D*, *TaCDPK20A*,*B*,*D*, *TaCDPK21B*,*D*, *TaCDPK27D*, and *TaCDPK29A*,*B*,*D* have been neglected due to their very low expression values (fragments per kilobase of exon per million mapped reads, FPKM < 0.1 or 0). Among the 79 members of *TaCDPK* family, *TaCDPK5A*, *TaCDPK7A*, *TaCDPK7B*, *TaCDPK25A*, *TaCDPK25B*, *TaCDPK26A*, *TaCDPK26B*, and *TaCDPK27B* showed higher FPKM value in most of the examined time points in the incompatible set of interaction (32R) compared to the compatible interaction (32S), suggesting their potential role in wheat resistance against *Pst* infection. Conversely, six members, *TaCDPK3A*, *TaCDPK3D*, *TaCDPK11A*, *TaCDPK22A*, *TaCDPK22B*, and *TaCDPK22D* from cluster I and cluster III, displayed the strongest induction during the compatible interaction, but were consistently downregulated at all examined time points during the incompatible interaction) (Figure 2), suggesting their possible negative roles in wheat defense against *Pst*.

### 2.4. Transcript Profile of TaCDPKs Challenged with Pst (qRT-PCR Analysis)

**Figure 3:**
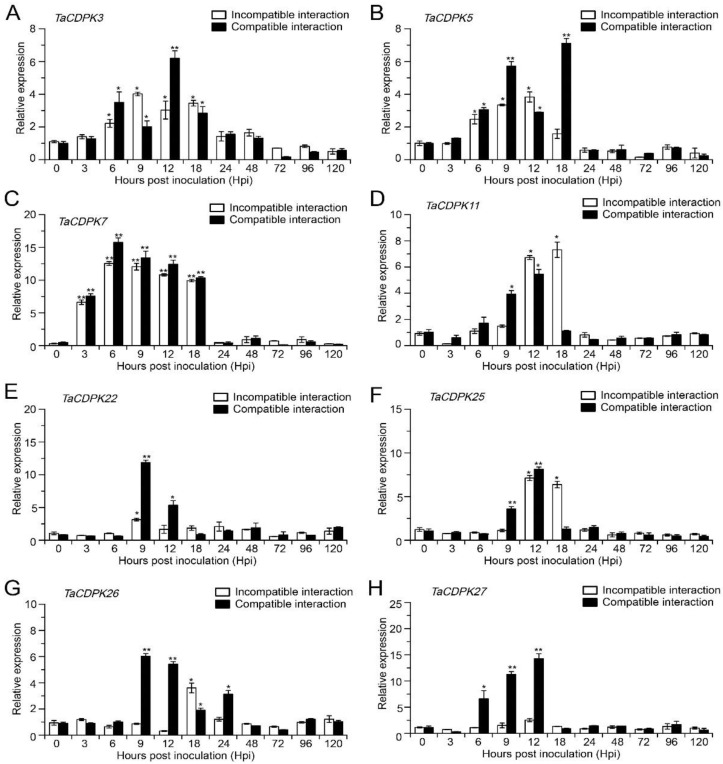
Expression profiles during the interaction of wheat with *Pst*. The relative expression of (**A**) *TaCDPK3*, (**B**) *TaCDPK5*, (**C**) *TaCDPK7*, (**D**) *TaCDPK11*, (**E**) *TaCDPK22*, (**F**) *TaCDPK25*, (**G**) *TaCDPK26*, and (**H**) *TaCDPK27* assessed at 0, 6, 12, 24, 48, and 120 hpi in leaf samples infected with *Pst* isolates CYR23 (incompatible interaction) and CYR31 (compatible interaction). Error bars represent the error or uncertainty in three independent replicates. qRT-PCR values were normalized to that of *TaEF-1α*. *p* values between different time points were assessed with student’s *t*-test (*, *p* < 0.05; **, *p* < 0.01).

To validate the RNA-seq data by qRT-PCR analysis, fourteen members of the *TaCDPK* family have been selected based on their expression level during compatible or incompatible interactions. These selected members are *TaCDPK3A*, *TaCDPK3D*, *TaCDPK5A*, *TaCDPK7A*, *TaCDPK7B*, *TaCDPK11A*, *TaCDPK22A*, *TaCDPK22B*, *TaCDPK22D*, *TaCDPK25A*, *TaCDPK25B*, *TaCDPK26A*, *TaCDPK26B*, and *TaCDPK27B*. Due to the high sequence similarity among homologous genes from different sub-genomes, such as *TaCDPK3A*/*TaCDPK3D* (97.27%), *TaCDPK7A*/*TaCDPK7B* (92.35%), *TaCDPK22A*/*TaCDPK22B*/*TaCDPK22D* (95.00%), *TaCDPK25A*/*TaCDPK25B* (98.25%), and *TaCDPK26A*/*TaCDPK26B* (97.72%), we performed qRT-PCR analysis using one representative gene copy from each pair. *TaCDPK3*, member of cluster III, showed strong significant upregulation at the middle stage 12 hpi of the compatible interaction (CYR31) compared with the mock control, as indicated in the RNA-seq data (Figure 2). Similarly, *TaCDPK5* from cluster I exhibited higher upregulation at 18 hpi during the compatible interaction (Figure 3), which is consistent with the RNA-seq data showing upregulation at the middle stage (24 hpi) of the compatible interaction (Figure 2). *TaCDPK11* displayed peak expression at middle stages (12 and 18 hpi) during the incompatible interaction (CYR23), followed by decreased expression in both compatible and incompatible interactions. Transcript levels of *TaCDPK22* showed significant upregulation at 9 and 12 hpi during the compatible interaction (Figure 3), which aligned with the RNA-seq data showing strong induction of *TaCDPK22* expression during all the time points of compatible set. Similar to the RNA-seq data, the mRNA accumulation of *TaCDPK25* showed significant upregulation at the middle stages (12 and 18 hpi) under the incompatible interaction (CYR23) (Figure 3), suggesting a positive role of *TaCDPK25* in wheat resistance against *Pst*. *TaCDPK26* displayed significantly higher expression during the compatible interaction at 9 and 12 hpi compared to the control (Figure 3). *TaCDPK27*, consistent with RNA-seq data showing strong expression during the compatible set, also showed significant upregulation at 6, 9, and 12 hpi during the compatible interaction (CYR31) (Figure 3) in qRT-PCR analysis, suggesting its possible role in wheat susceptibility to *Pst*. *TaCDPK7* exhibited strong induction at most of the time points (3, 6, 9, 12, 18 hpi) in both compatible and incompatible interactions (Figure 3). Both the RNA-seq and qRT-PCR results suggest that *TaCDPK7* may play a potential role in wheat–*Pst* interaction, either positively or negatively. Therefore, *TaCDPK7* was selected for further investigation of its functional role in wheat defense against *Pst*. Overall, our results indicate the reliability of the RNA-seq data, and highlight the regulation of several *TaCDPK*s during the wheat–*Pst* interaction.

### 2.5. Silencing of TaCDPK7 Enhances Wheat Susceptibility to Pst

**Figure 4:**
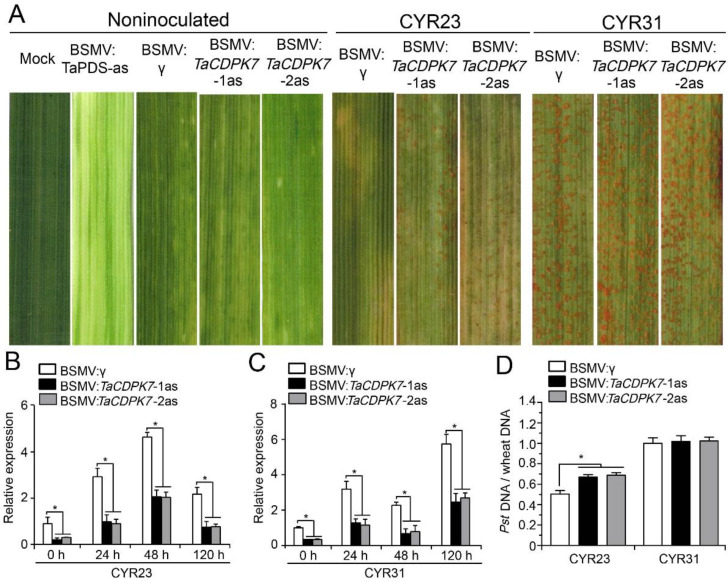
Silencing of *TaCDPK7* enhances wheat susceptibility to *Pst.* (**A**) Mild chlorotic mosaic symptoms on viral-inoculated wheat plants. Mock: wheat leaves treated with 1 × Fes buffer. The leaves infected with the avirulent race CYR23 or the virulent race CYR31 were photographed at 14 days post infection (dpi). (**B**,**C**) Transcripts of *TaCDPK7* in *TaCDPK7*-knockdown or control plants (plants inoculated with BSMV:γ) challenged with the avirulent CYR23 and virulent CYR31. (**D**) The fungal/wheat biomass ratio of *TaCDPK7*-knockdown and control plants at 7 dpi (*, *p* < 0.05).

To investigate the functional role of *TaCDPK7* during the wheat–*Pst* interaction, we employed BSMV-gene silencing, a widely used and effective tool in wheat–*Pst* interaction studies [30,31]. Two specific fragments within the coding region of *TaCDPK7* were selected to silence its transcripts. Wheat seedling leaves were inoculated with BSMV:*TaPDS*-as, BSMV:*TaCDPK7*-1as, BSMV:*TaCDPK7*-2as, and BSMV:γ. Leaves inoculated with BSMV:*TaPDS* exhibited strong photo-bleaching symptoms (Figure 4A), while mild chlorotic mosaic patterns were observed on leaves infected with BSMV:γ, *TaCDPK7*-1as, and *TaCDPK7*-2as, confirming the functionality of the gene silencing system.

The fourth leaf of wheat plants pre-inoculated with BSMV:γ, *TaCDPK7*-1as, or *TaCDPK7*-2as was subsequently inoculated with the avirulent *Pst* isolate CYR23 or the virulent isolate CYR31 at 10 days post inoculation (dpi). At 14 days after inoculation with CYR23, clear hypersensitive responses (HRs) were observed on all the virus-pre-inoculated leaves (Figure 4A). Fungal uredia were produced near the necrotic areas on the leaves of *TaCDPK7*-silenced plants but not on leaves pre-inoculated with BSMV (Figure 4A), indicating that silencing of *TaCDPK7* reduced the resistance of wheat to CYR23. Conversely, normal disease development was observed on CYR31-inoculated plants, and no significant difference was observed between the control (BSMV:γ-pre-inoculated) and the *TaCDPK7*-silenced plants (Figure 4A).

To confirm the effective silencing of *TaCDPK7*, the transcript levels of *TaCDPK7* were analyzed via qRT-PCR in leaves inoculated with BSMV:*TaCDPK7*-1as and BSMV:*TaCDPK7*-2as. The transcript levels in *TaCDPK7*-knockdown plants from both incompatible and compatible interactions were significantly reduced up to two-fold compared to those in control plants, indicating successful silencing of *TaCDPK7* (Figure 4B,C). Furthermore, the *TaCDPK7*-knockdown plants exhibited a significantly increased fungal biomass compared to the control plants during the incompatible interaction, while no significant difference in fungal biomass ratio was detected between the silenced and control plants during the compatible interaction (Figure 4D). Collectively, these results demonstrated that silencing of *TaCDPK7* compromises the resistance of wheat to the stripe rust isolate CYR23.

### 2.6. Knockdown of TaCDPK7 Declines ROS Accumulation

**Figure 5:**
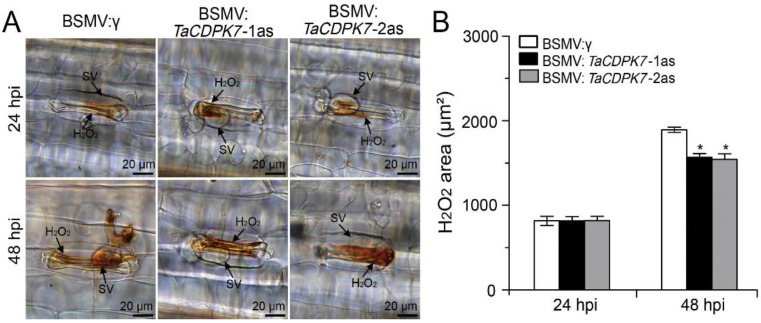
Silencing of *TaCDPK7* decreased H_2_O_2_ accumulation. (**A**) H_2_O_2_ accumulation and fungal structure in wheat leaves inoculated with *Pst* race CYR23 after being pre-infected with BSMV:γ, *TaCDPK7*-1/2as at 24 and 48 hpi. H_2_O_2_ accumulation was examined with DAB (3,3′-diaminobenzidine) staining. SV, sub-stomatal vesicle. (**B**) H_2_O_2_ accumulation assessed by quantifying the DAB-stained area. Asterisks show significant differences between *TaCDPK7*-knockdown and control plants, as determined by a student’s *t*-test (*, *p* < 0.05).

Since CDPKs have been implicated in ROS (reactive oxygen species) production in *Arabidopsis* and *Solanum tuberosum*, we further investigated the accumulation of H_2_O_2_, which is strongly associated with the host resistance response, in *TaCDPK7*-silenced plants during the wheat–*Pst* incompatible interaction. Interestingly, the production of H_2_O_2_ in *TaCDPK7*-silenced plants was significantly reduced compared to control plants treated with BSMV:γ at 48 hpi, although there was no difference between them at 24 hpi (Figure 5A,B). These findings suggest that TaCDPK7 may also play a role in regulating H_2_O_2_ production in wheat in response to *Pst* infection.

### 2.7. Knockdown of TaCDPK7 Improves Pst Growth

**Figure 6:**
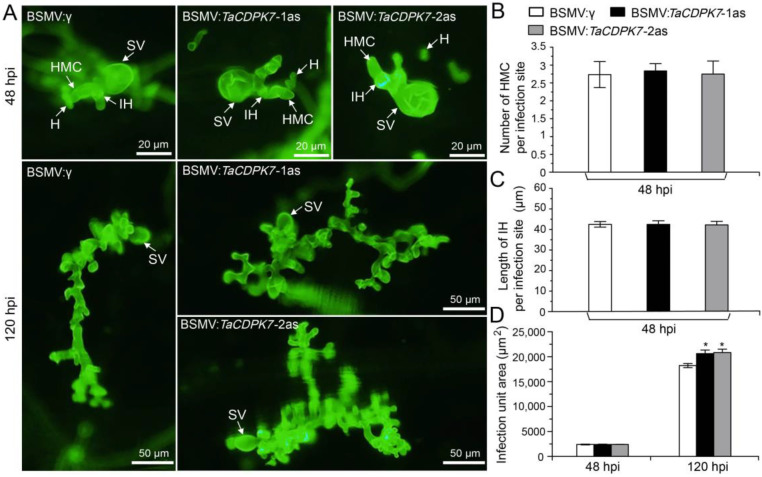
Knockdown of *TaCDPK7* enhanced pathogen growth. (**A**) Pathogen advancement per infection site. Number of (**B**) haustorium mother cells (HMCs), (**C**) infection hypha (IH), and (**D**) infection area per site were measured in silenced and control plants. Asterisks show significant differences between *TaCDPK7*-knockdown and control plants, as determined by a student’s *t*-test (*, *p* < 0.05).

To assess whether the reduced resistance in *TaCDPK7*-knockdown plants was due to the limited growth of *Pst* in the plants, we conducted histological observations of wheat leaves during the incompatible interaction. At 48 hpi, there was no significant difference in hyphal growth, as indicated by the number of haustoria mother cells per infection site, hyphal length, and infection unit area, between the leaves of the control plants and those of the silenced plants (Figure 6A–D). However, at 120 hpi, the infection unit area was significantly larger in the *TaCDPK7*-silenced leaves compared to the controls (Figure 6A,D), suggesting that silencing the transcription of *TaCDPK7* enhanced fungal growth in wheat leaves.

### 2.8. Transcript Levels of PR and ROS-Related Genes Are Affected in TaCDPK7-Silenced Plants

**Figure 7:**
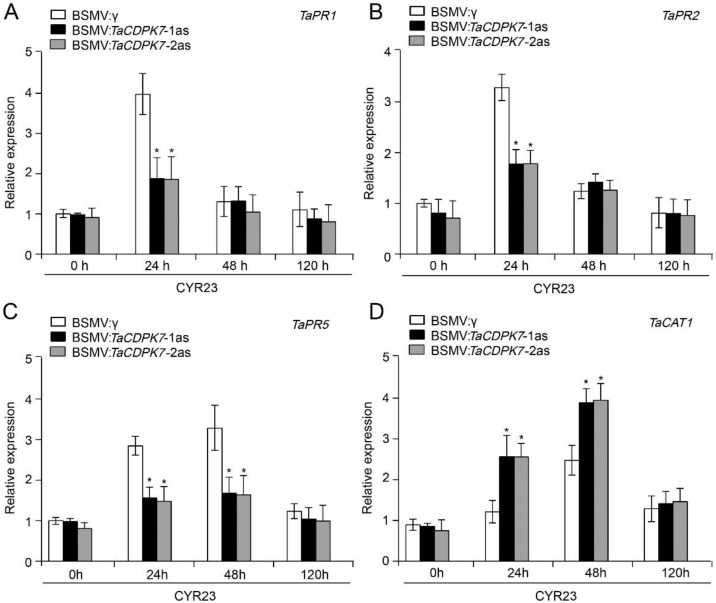
Transcriptional changes in pathogenesis-related (PR) genes and ROS-scavenging gene *TaCAT1* in *TaCDPK7*-silenced and control plants challenged with avirulent *Pst* race *CYR23.* Relative expressions of (**A**) *TaPR1*, (**B**) *TaPR2*, (**C**) *TaPR5*, and (**D**) *TaCAT1* were analyzed by qRT-PCR and the comparative threshold (2^−ΔΔCT^) method. Asterisks show significant differences between *TaCDPK7*-knockdown and control plants as assessed by a student’s *t*-test (*, *p* < 0.05). All data were attained from three biological replicates.

To assess the impact of *TaCDPK7* silencing on the expression of PR-related and ROS-scavenging genes, we analyzed the transcript levels of three *PR* genes (*TaPR1*, *TaPR2*, and *TaPR5*) and one ROS-scavenging gene (*TaCAT1*, catalase) using qRT-PCR. The Arabidopsis CAT1, CAT2, and CAT3 are crucial for the elimination of H_2_O_2_, and play important roles in regulating ROS homeostasis. Notably, the AtCAT1 specifically targets the removal of H_2_O_2_ generated under various environmental stress conditions [32]. Therefore, *TaCAT1*, the wheat homolog of *AtCAT1*, was selected for examination in this study. In *TaCDPK7*-silenced plants, the expression of *PR* genes (*TaPR1*, *TaPR2*, and *TaPR5*) were significantly downregulated compared to the control plants, both in incompatible and compatible interactions (Figure 7 and Figure S6). Conversely, the transcripts of the ROS-scavenging gene (*TaCAT1*) were significantly increased in *TaCDPK7*-silenced plants compared to the control plants during both incompatible and compatible interactions (Figure 7 and Figure S6). These findings indicate that *TaCDPK7* acts as a positive regulator of certain defense-related genes during the wheat–*Pst* interaction.

## 3. Discussion

CDPKs are essential calcium sensors with calcium binding and signal transducing capabilities, playing crucial roles in plant development and stress responses. While genome-wide studies on CDPK families have been conducted in several plants such as rice [27], Arabidopsis [3], and barley [33], limited research has focused on CDPKs in bread wheat, a vital food source for humans. Earlier studies identified 20 CDPKs in wheat and observed their expression patterns under various stresses in 2008, when the wheat genome was not fully sequenced [22]. The hexaploid wheat originated through complex evolutionary processes approximately 8000–10,000 years ago [34]. It underwent two rounds of genome duplication, leading to the formation of a complex genome comprising three linked sub-genomes (BB, AA, DD). These sub-genomes originated from three distinct diploid species [34].

Therefore, it is expected that hexaploid wheat possesses a larger number of CDPK genes due to its complex genome resulting from the combination of three distinct sub-genomes. Consistently, we identified 79 CDPK genes from the hexaploid wheat genome, which is a higher number compared to the number of *CDPK*s in rice and *Arabidopsis*. This observation further supports the expectation of a larger repertoire of *CDPK* genes in hexaploid wheat due to its complex genome structure. Phylogenetic analysis grouped wheat *CDPK*s into four clusters (I-IV), similar to rice *CDPK*s [27]. All identified *CDPK* genes (26 + 26 + 26) are equally distributed across sub-genomes A, B, D, except for *TaCDPK4UN*, which is located on an unknown locus (Table S1). All the *TaCDPK*s have counterparts in the other two sub-genomes, except for *TaCDPK18* and *TaCDPK27*, they lack copies in sub-genome B and A, respectively, which is possibly due to genetic erosion. Moreover, possible tandem duplication and segmental duplication events were observed among *TaCDPK*s. These findings suggest that the variations in the number of CDPK family members among different plants are likely a result of gene loss and gene duplication, which are key factors in the evolution of gene families. Such evolutionary processes can lead to the emergence of new genes with distinct roles [35]. Consistent with the phylogenetic relationships among species, the wheat CDPKs exhibit a closer phylogenetic association with that of rice CDPKs in comparison with that of *Arabidopsis*, and most *Arabidopsis* CDPK members exhibited a higher degree of divergence from the other two taxa (wheat and rice). This observation might be attributed to the fact that both wheat and rice (monocot) belong to the same Poaceae family in contrast to *Arabidopsis*, which is a dicot belonging to the family Brassicaceae.

CDPKs are membrane-embedded proteins, and their second signal’s reversibility as calcium sensors may allow CDPKs to shuttle between membranes and the cytosol or the nucleus. Here, we also predicted a diverse range of cellular localizations of CDPKs, including the cytosol, chloroplasts, plasma membrane, nucleus, endoplasmic reticulum, mitochondria, and peroxisomes (Table S4), suggesting that TaCDPKs have access to a large range of possible substrates throughout the cell and have a wide variety of functions. CDPKs are evolved in perceiving changes in Ca^2+^ levels within plant cells and play a crucial role in disease resistance and stress-responsive signaling pathways. These proteins decode and interpret Ca^2+^ flux signals through phosphorylation processes, which are crucial events for signal transduction pathways. Numerous functional and expression studies of different *CDPK*s confirmed their versatile roles in drought, salt, cold stress tolerance, as well as calcium homeostasis in plant cells. Additionally, CDPK family members from diverse plant species have been reported to be implicated in disease defense mechanism. The *Arabidopsis* CDPKs AtCPK4/5/6/11 phosphorylate the transcription factors WRKY8/28/48, thereby modulating the expression of downstream biotic-responsive genes [36,37]. In particular, AtCPK4/5/6/11 from group I of *Arabidopsis* CDPK family classification induced flg22-mediated gene expression and production of ROS [19,38]. They target RBOHD on phosphosites S148/S163/S347 that are PAMP stimulated and required for RBOHD activity [38,39,40,41]. Interestingly, a fifth isoform from subgroup IV, AtCPK28, negatively regulates PAMP-induced oxidative burst by reducing the stability of another RBOHD-stimulating enzyme *Botrytis*-induced kinase1 (BIK1) [42]. AtCPK5/6 also regulate ethylene production in response to wounding and *Botrytis. cinerea* infection, through the modulation of ethylene biosynthesis enzyme ACC synthase (ACS), either at the transcriptional level or by stabilizing the proteins [43,44]. CPKs from subgroups II and III have also been involved in plant immunity. In particular, *AtCPK3* was implicated in various defense pathways. It was required for phytosphingosine-induced PCD in response to the fungus *Fusarium moniliforme* [45]. There are few reports of CDPKs role against plant viruses resistance, in which AtCPK3 could limit cell-to-cell propagation of potato virus X by phosphorylating the remorin StRem1.3 [46]. In a recent study, TaTHI2 interacts with TaCPK5 to suppress Chinese wheat mosaic virus (CWMV) infection [47]. Despite previous evidence showcasing the positive role of wheat *TaCPK7-D*, assigned to subgroup III based on previous wheat CDPK family classification, in enhancing resistance against *Rhizoctonia cerealis* [48], there is limited research on the activities of wheat CDPKs during biotic stress. In this study, we showed that several *TaCDPK*s were either upregulated or downregulated during the compatible and incompatible interactions between wheat and *Pst*, as shown in Figure 2, suggesting that certain *TaCDPK*s may potentially contribute to wheat defense against *Pst*. Specifically, *TaCDPK7*, a member of cluster I, displayed strong induction in both compatible and incompatible interactions (Figure 3), highlighting its potential importance in the defense response of wheat against *Pst*.

In barley and wheat, BSMV-mediated VIGS has been established as a quick and effective reverse genetic technique for studying gene functions [49]. Silencing of *TaCDPK7* by VIGS caused increased susceptibility to *Pst* accompanied by an enlarged infection area within leaf tissues in *TaCDPK7*-silenced plants (Figure 6), strongly suggesting that *TaCDPK7* plays a crucial role in conferring wheat resistance to *Pst*. The ROS burst, one of the earliest signaling events in plants, happens in the initial phases during plant–pathogen interactions [50]. As demonstrated in this study, the ROS accumulation was significantly reduced in *TaCDPK7*-knockdown plants. Furthermore, the transcript levels of the ROS-scavenging gene (*TaCAT1*) were significantly induced in *TaCDPK7*-knockdown plants related to control plants in incompatible and compatible interactions (Figure 7 and Figure S6). These findings suggest that *TaCDPK7* plays a role in wheat’s resistance against *Pst* by inhibiting the expression of the ROS-scavenging gene *TaCAT1*, thereby leading to increased ROS content in wheat. Plant defense responses to pathogens are accomplished through transcriptional activation of defense-related genes. The overexpression of *OsCDPK1* has been demonstrated to upregulate the rice *PATHOGEN-RELATED 1/4/10a* (*PR1*, *PR4*, and *PR10a*) genes, and thereby positively affecting rice resistance to *Xoo* infection [51]. In alignment with these findings, our study observed a downregulation of transcript levels for *PR* genes *TaPR1*, *TaPR2*, and *TaPR5* in *TaCDPK7*-knockdown plants during both incompatible and compatible interactions (Figure 7 and Figure S6). Overall, our data strongly indicate that *TaCDPK7* plays a positive role during wheat resistance to *Pst* by promoting ROS accumulation and enhancing the expression of *PR* genes. However, to further strengthen and solidify this conclusion, it would be valuable to conduct future investigations focusing on the effects of *TaCDPK7* overexpression during the wheat–*Pst* interaction, which would provide a complementary approach to validate and expand upon the findings presented in this study, ultimately enhancing our understanding of the precise role of *TaCDPK7* in wheat resistance to *Pst*.

## 4. Materials and Methods

### 4.1. Plant Material and Fungal Isolates

Wheat cultivar Suwon 11 (Su11) and *Pst* isolates CYR23 (avirulent), and CYR31/CYR32 (virulent) were used in this study. Wheat cultivar Su11 and *Pst* isolates, CYR23, CYR31, and CYR32 were obtained from the Institute of Plant Pathology, Northwest A&F University. Su11 is reported to be resistant to CYR23 but susceptible to CYR31 and CYR32 [52]. The wheat cultivars NIL_R (*Yr*26) and NIL_S (*yr*26) were generated as described in [53]. All the plants in this study were grown in a growth chamber (Percival, Des Moines, IA, USA) under a controlled environment at 14–16 °C, half lamps, with a 16 h photoperiod.

### 4.2. RNA, DNA Extraction, and cDNA Synthesis

The wheat cultivar Suwon 11 was infected with *Pst* race CYR23 (avirulent), CYR31 (virulent), or CYR32 (virulent). For RNA extraction, *Pst* inoculated samples were collected at 0, 6, 12, 24, 48, 72, and 120 hpi (hours post inoculation) [54,55]. Then, total RNA was extracted with the Quick RNA isolation Kit (Huayueyang Biotechnology, Beijing, China) following the manufacturer’s instructions and contaminating DNA was removed by treatment with DNase I enzyme. First-strand cDNA was synthesized using 2 μg of the extracted total RNA with the Promega Reverse Transcription System (Promega, Madison, WI, USA) and Oligo (dT) 18 primer. To assess the fungal/wheat biomass ratio, samples were collected at 7 dpi (days post inoculation) for genomic DNA extraction. Quantitative PCR of the DNA was conducted relative to the internal reference genes *PstEF-1* and *TaEF-1a*, respectively, according to the previous study [56].

### 4.3. Mining of CDPKs from Wheat Genome

To identify the putative *CDPK*s in wheat, a local BLASTP search in the wheat database (http://plants.ensembl.org/ (accessed on 6 September 2022)) was performed with e-value of ≤e^−10^. Two BLAST approaches were implemented to search the wheat CDPK proteins. First, all known *Arabidopsis* CDPK protein sequences retrieved from TAIR (www.arabidopsis.org (accessed on 17 July 2022) were used as query to search their corresponding potential homologs in the wheat genome. Secondly, the similar method was performed using all known rice CDPK sequences retrieved with NCBI (https://www.ncbi.nlm.nih.gov/ (accessed on 3 August 2022)). All the matching sequences were further validated with the SMART program and InterPro, an online catalogue for protein classification (https://www.ebi.ac.uk/interpro/ (accessed on 15 October 2022)), with default settings. The protein sequences lacking the signature domains were excluded from further analysis. As a result, 79 identified CDPK candidates with the conserved domains were designated as TaCDPK proteins.

### 4.4. Phylogeny and Gene Structure Analysis

Maximum likelihood mid-rooted phylogenetic tree was built using the mega 6 tool to investigate the evolutionary relationships between CDPKs in wheat, *Arabidopsis*, and rice. Afterwards, the data of the corresponding tree were uploaded at Interactive Tree of Life (IToL) v. 6 (https://itol.embl.de/ (accessed on 30 September 2022)), an online program and a phylogenetic tree of wheat CDPKs was drawn with scale bars corresponding to 0.1 substitution, as previously reported [57,58]. The exon–intron assemblies of *TaCDPK*s were analyzed with Gene Structure Display Server v. 2.0 as described in [59].

### 4.5. The Conserved Motif, Cis-Acting Regulatory Elements, and Synteny Analysis

The conserved motifs of TaCDPKs were analyzed using MEME (multiple expectation maximization for motif elicitation) v. 5.3 with default parameters as described by [60]. Amino acid sequences of TaCDPKs ranging from 6 to 100 residues in length were used for identifying conserved motifs. The subcellular localization of TaCDPK proteins was predicted using the online program WOLFPSORT II, with default options and selecting plants as the organism type, following the method by Waterhouse et al. [61]. Biochemical characteristics of TaCDPK proteins, such as molecular weight (MW), amino acid composition, instability index, theoretical pI, aliphatic index, and grand average of hydropathicity (GRAVY), were determined using the ExPASy bioinformatics resource portal. Default options were selected for the analysis, following the protocol described by Artimo et al. [62]. To explore the potential regulatory mechanisms of *TaCDPK* genes, the 1.5 kb upstream promoter region of each gene was extracted from the wheat genome reference database. The *cis*-elements of *TaCDPK* promoters were assessed using the plant *cis*-acting regulatory DNA elements (PLACEs) and plant *cis*-acting regulatory element (PlantCARE) tools, following the methods previously employed [63,64]. For synteny illustration, TBtools data managing toolkit by Chen et al. [65] was utilized.

### 4.6. Expression Profiling of TaCDPK Family Challenged with Pst

The transcript levels of all the *TaCDPK* genes during wheat *Pst* compatible (NIL_R vs. CYR32) and incompatible (NIL_S vs. CYR32) interactions were examined using RNA-seq data obtained from wheat samples collected at 0, 18, 24, 48, 96, and 168 hpi as previously described [66]. Each sample was sequenced with HiSeq2500 (PE125) as described in [67]. Corresponding heat maps demonstrating the transcript levels of *TaCDPKs* were generated using log2^FC^ by an online program (https://software.broadinstitute.org/morpheus/ (accessed on 20 February 2023)).

### 4.7. Real-Time PCR (qRT-PCR) Analysis

Total RNAs were extracted with the Trizol reagent according to the manufacturer’s protocols (Invitrogen, Carlsbad, CA, USA), and then treated with DNase I (Promega, Madison, WI, USA) to remove DNA contamination. cDNA was synthesized with GoScript Reverse Transcription System (Promega, USA) and an oligo (dT18) primer (Invitrogen, USA). Primers specific to the examined TaCDPKs were used to perform qRT-PCR using the synthesized cDNA as a template (Table S3). A 7500 Real-Time PCR System (Applied Biosystems, San Francisco, CA, USA) was used to quantify the transcripts. *TaEF-1α* (GenBank Q03033) was used as the internal reference gene. The relative expression of *TaCDPKs* was determined with comparative method 2^−ΔΔCT^ [68], qRT-PCR experiments were carried out three times.

### 4.8. BSMV-Mediated Gene Silencing of TaCDPK7

Silencing of *TaCDPK7* was achieved using the barley stripe mosaic virus (BSMV)-mediated gene silencing method, as previously described [49]. Two cDNA fragments of *TaCDPK7* were selected and incorporated into the BSMV-γ-vector, resulting in the construction of BSMV-γ:*TaCDPK*7-1as/2as constructs. Capped in vitro transcripts were generated using the RiboMAXTM (LargeScale RNA Production System-T7, Promega, Madison, WI, USA) and Ribom7G Cape Analog (Promega, Madison, WI, USA) according to the manufacturer’s protocol.

The barley stripe mosaic virus constructs were inoculated onto the second leaf of the wheat plants, following the previously reported method [49]. The plants were then kept in darkness at a temperature of 25 ± 2 °C for 24 h with adequate humidity. Control plants were inoculated with 1 × Fes buffer (0.06 M K2HPO4, 0.1 M glycine, 1% *w*/*v* tetrasodium pyrophosphate, 1% *w*/*v* celite, 1% *w*/*v* bentonite, pH 8.5). After 10 days, fresh urediniospores of *Pst* race CYR23 or CYR31 were collected and inoculated onto the fourth leaf. Leaf samples treated with *Pst* were collected at 0, 24, 48, and 120 hpi for histological investigation and RNA extraction. The silencing efficacy of the *TaCDPK*7-silenced plants and the relative expression of pathogenesis (*PR*) and ROS-related genes were quantified using qRT-PCR analysis. After 14 days, the phenotypic expression of *Pst*-inoculated leaves was assessed, and infection phenotypes were documented through photography. The entire experiment was repeated three times.

### 4.9. Histology of Fungal Infection and Host Response

Wheat leaves were collected at 24, 48, and 120 hpi for the examination of H_2_O_2_ accumulation using 3,3′-diaminobenzidine (DAB) staining, following the method reported by Bai et al. [54]. To investigate fungal growth in planta, leaves collected at the same time points were stained with wheat germ agglutinin (WGA) conjugated to Alexa (Invitrogen, Carlsbad, CA, USA). The presence of a vesicle under a stoma was used as an indicator of infection sites and at least 30 infection sites per samples were examined for both H_2_O_2_ accumulation and fungal growth investigation. The H_2_O_2_ accumulation and infection area were visualized using an Olympus BX-53 microscope (Olympus Corporation, Tokyo, Japan) and quantified using DP-BSW Ver. 03. 03 software.

### 4.10. Statistical Analysis and Graphical Presentation

All data were analyzed with GraphPad Prism v. 7 software. Student’s *t*-test was employed to determine the statistical differences between treatments.

## 5. Conclusions

In summary, our genome-wide investigation identified a total of 79 *CDPK* genes in hexaploid wheat. Among these genes, several displayed differential expressions during the wheat–*Pst* interaction, with *TaCDPK7* exhibiting the strong significant induction. Further functional characterization revealed that *TaCDPK7* plays a crucial role in conferring wheat resistance against *Pst* by curbing fungal growth in leaf tissues. Gene expression analysis indicated that *TaCDPK7* contributes to wheat defense against *Pst* infection by promoting the expression of *PR* genes and downregulating the transcription of the ROS-scavenging gene *TaCAT1*. The findings of this study not only provide a promising candidate gene for enhancing wheat resistance to *Pst*, but also shed light on the role of the *CDPK* family during biotic stress. Additionally, this study serves as a valuable reference for identifying key genes that can be targeted for breeding purposes to improve pathogen resistance in wheat.

## Data Availability

Data are contained within the article and Supplementary Materials.

## Supplementary Materials

The following supporting information can be downloaded at: https://www.mdpi.com/article/10.3390/ijms25021048/s1.
