# Supplementary material for: The Calcium-Dependent Protein Kinase TaCDPK7 Positively Regulates Wheat Resistance to Puccinia striiformis f. sp. tritici
Source: Int J Mol Sci. 2024 Jan 15;25(2):1048. doi: 10.3390/ijms25021048 (PMC10816280; doi:10.3390/ijms25021048)
Supplement: Supplementary file 1 [file ijms-25-01048-s001.zip › ijms-2775572-supplementary.pdf]

Supplementary Figures;

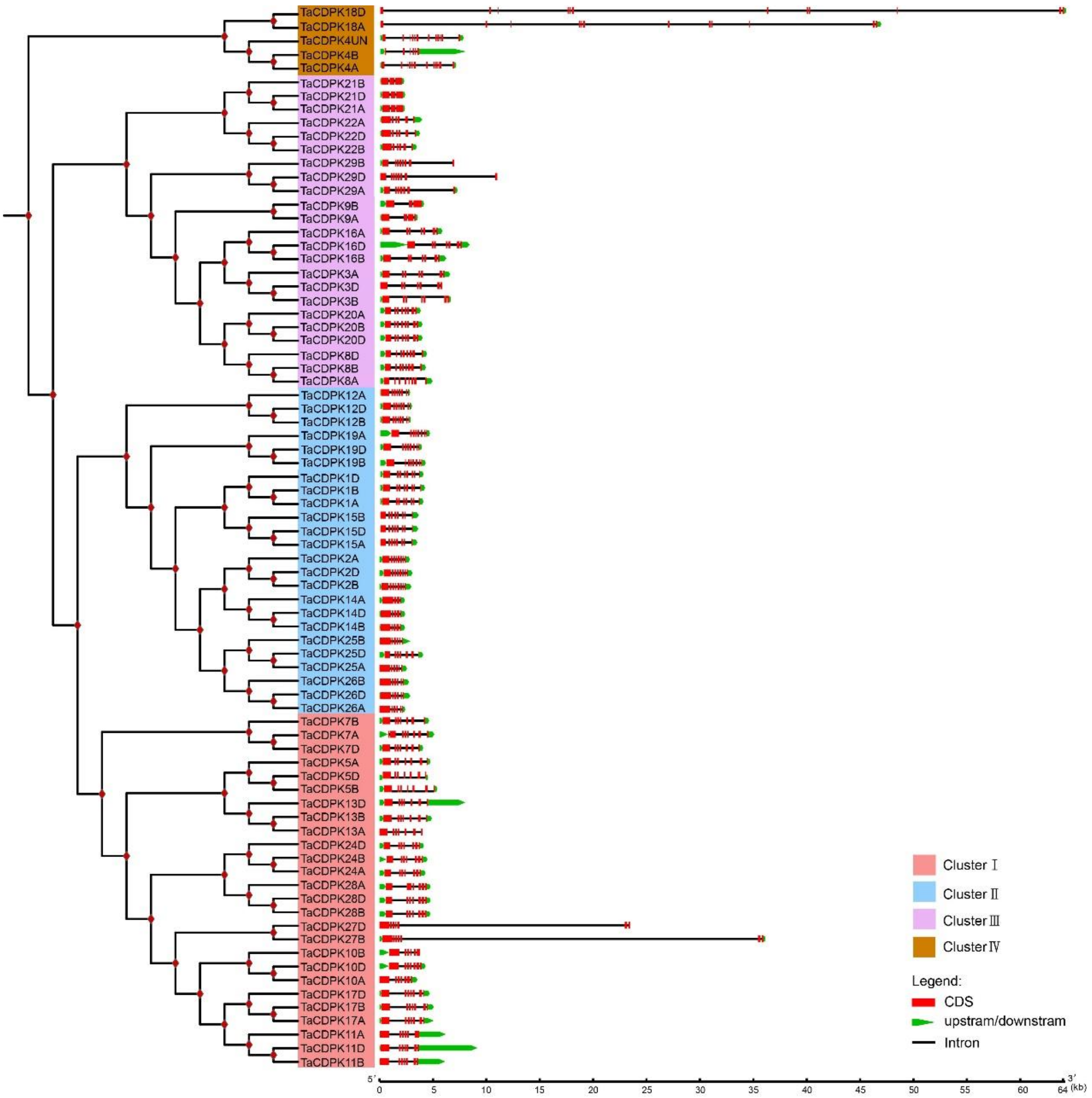

Figure S1. Gene structure of *TaCDPK*s. Exons are represented by red boxes, introns by black lines, and the UTR region by blue boxes.

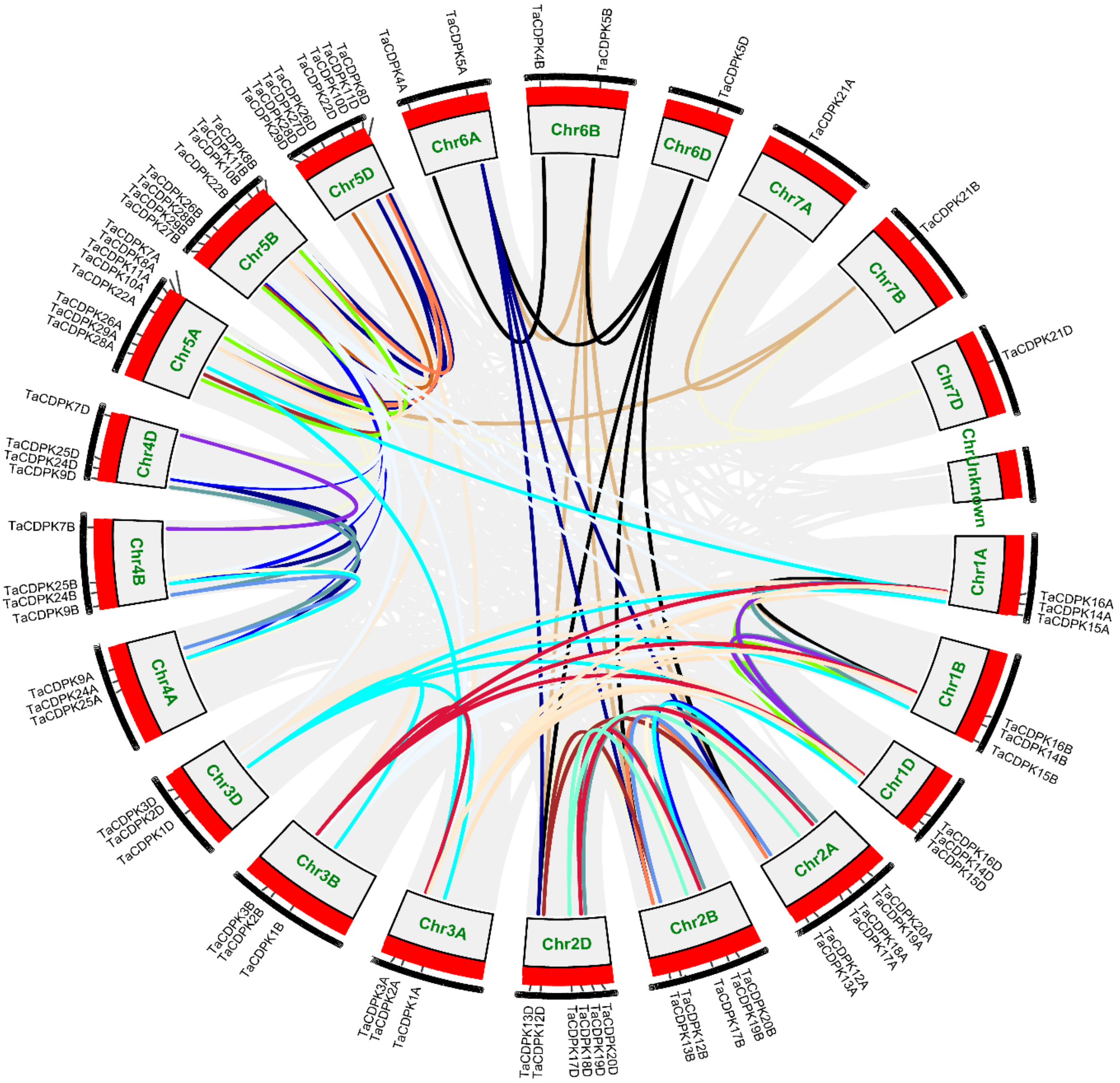

Figure S2. The synteny and chromosomal distribution of the *TaCDPK* genes. Colored bars connecting two chromosomal areas reflect syntenic regions; related genes on two chromosomes were considered segmental duplication.

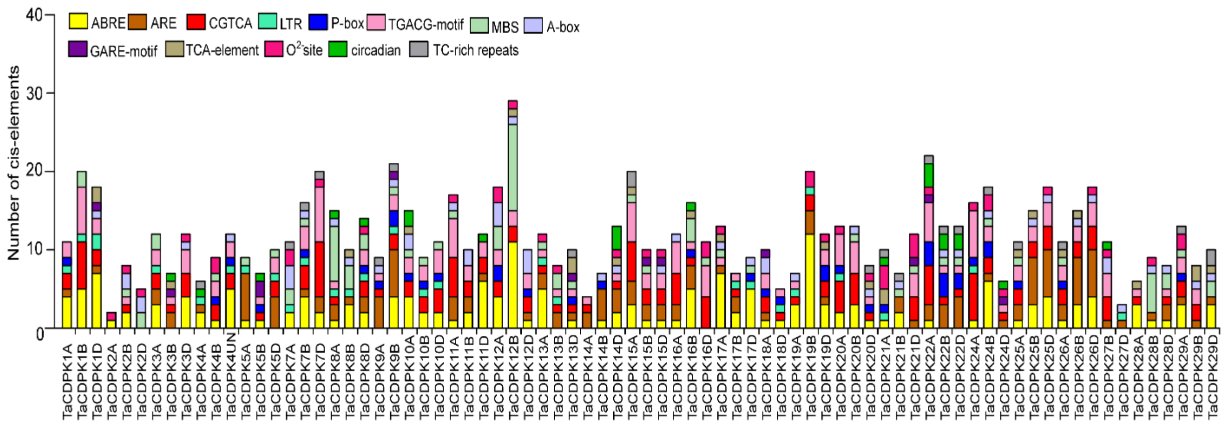

Figure S3. *Cis*-regulatory elements discovered in the 1.5 kb promoter of *TaCDPK* genes via an online server PlantCARE and PLACE. Different colors indicate *Cis* elements associated with various functions.

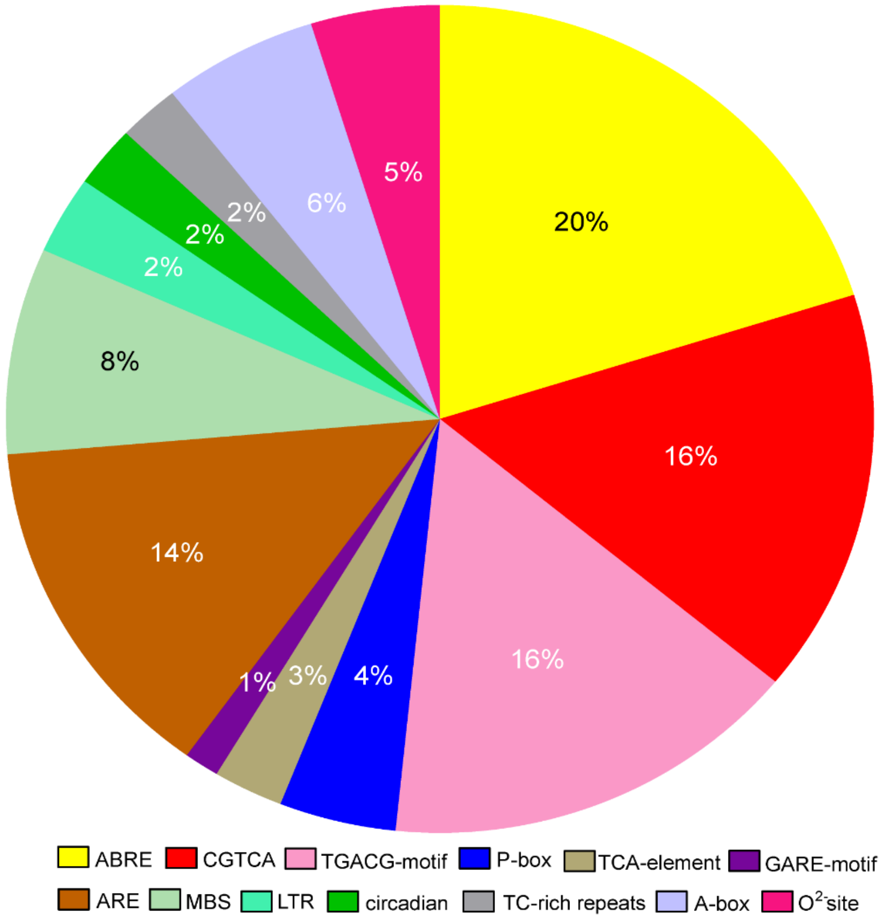

Figure S4. The percentage of the identified *cis*-elements in the entire *TaCDPK* family associated with their respective biological functions.

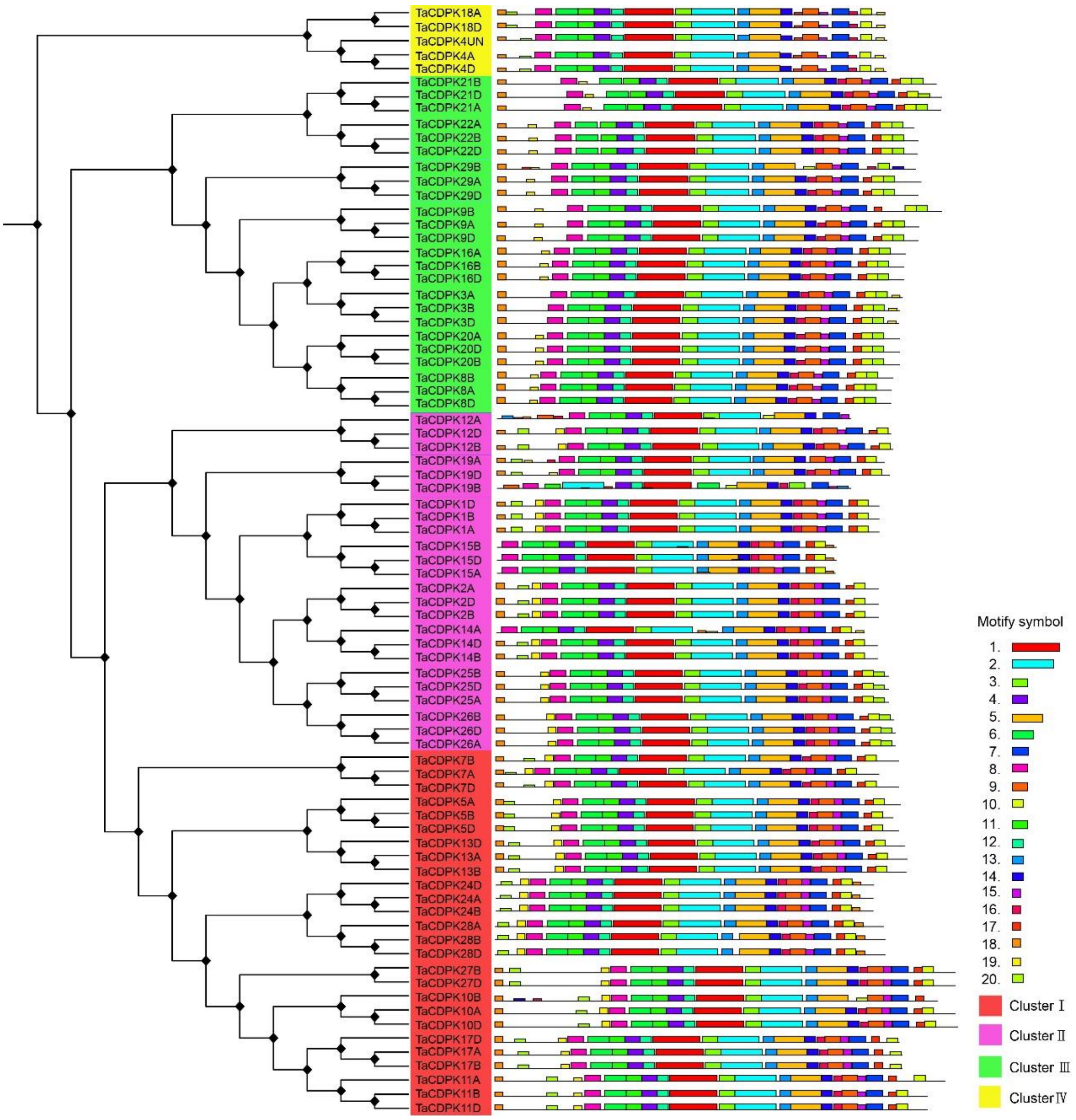

Figure S5. Phylogenetic relationships conserved motifs in TaCDPKs identified by MEME software. The identified motifs are represented by different colors. The length of the box represents the length of the motif.

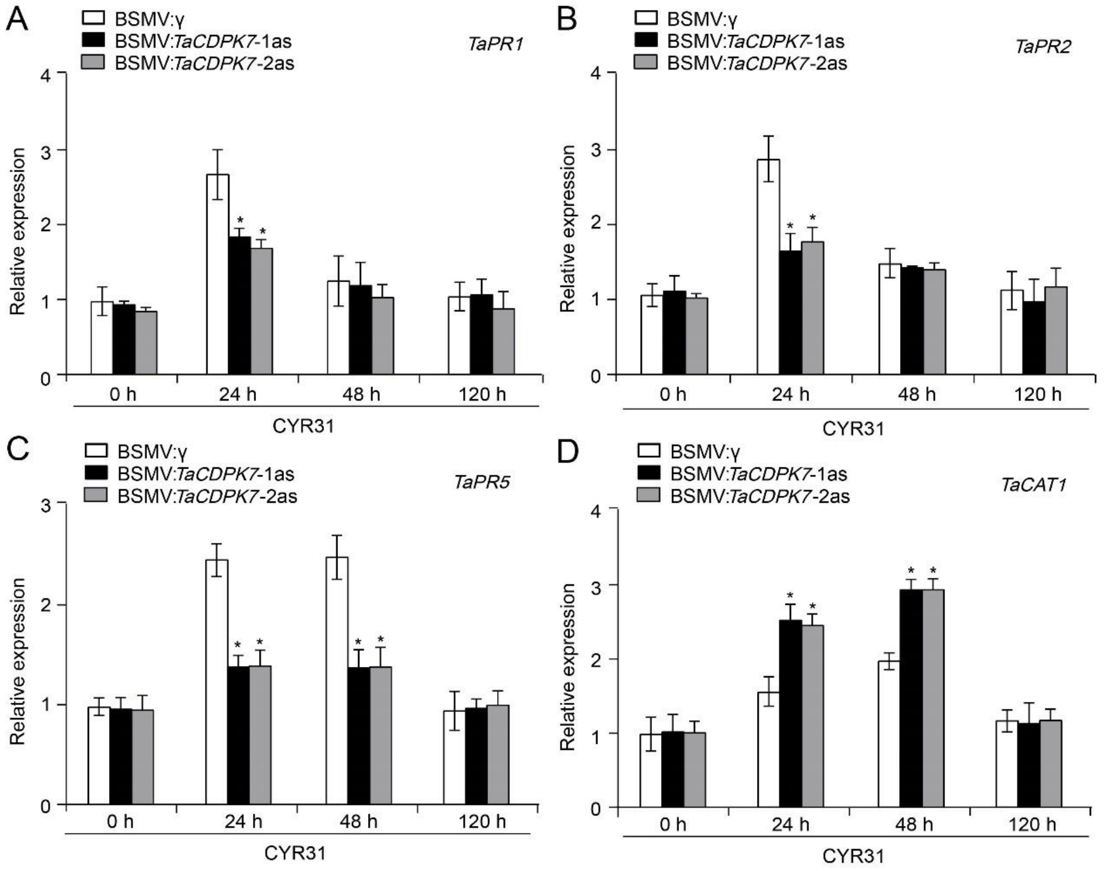

Figure S6. Transcripts level of pathogenesis related genes (*TaPR1*, *TaPR2*, *TaPR5*) and the ROS-scavenging gene *TaCAT1* in *TaCDPK7*-silenced and control plants challenged with *Pst* virulent race CYR31. (*, $P < 0.05$).

Supplementary Tables
Table S1
Characteristics of TaCDPKs

Accession No.	Gene ID	Chromosome No.	Start site	End site	CDS (bp)	ORF (aa)	Splice variant
TraesCS3A02G224900	*TaCDPK1A*	3A	421118493	421122535	1558	518	1
TraesCS3B02G254500	*TaCDPK1B*	3B	410040342	410044532	1558	518	1
TraesCS3D02G228700	*TaCDPK1D*	3D	310919760	310923812	1558	518	1
TraesCS3A02G334200	*TaCDPK2A*	3A	579552578	579555418	1558	518	1
TraesCS3B02G365200	*TaCDPK2B*	3B	576657225	576660207	1558	518	1
TraesCS3D02G327700	*TaCDPK2D*	3D	439838218	439841306	1558	518	3
TraesCS3A02G351300	*TaCDPK3A*	3A	599860801	599867349	1645	547	1
TraesCS3B02G383800	*TaCDPK3B*	3B	603601410	603608014	1630	542	1
TraesCS3D02G345500	*TaCDPK3D*	3D	457018710	457024515	1633	543	1
TraesCS6A02G089400	*TaCDPK4A*	6A	57725641	57732746	1537	511	2
TraesCS6B02G111800	*TaCDPK4B*	6B	93561956	93569973	1537	511	1
TraesCSU02G016900	*TaCDPK4UN*	UN	19226343	19234144	1540	512	2
TraesCS6A02G261200	*TaCDPK5A*	6A	484609229	484612543	1654	550	1
TraesCS6B02G288600	*TaCDPK5B*	6B	519721309	519725056	1648	548	1
TraesCS6D02G242000	*TaCDPK5D*	6D	344208648	344211808	1648	548	2
TraesCS5A02G490200	*TaCDPK7A*	5A	659722140	659727259	1567	521	2
TraesCS4B02G321800	*TaCDPK7B*	4B	612759007	612763642	1648	548	1
TraesCS4D02G318400	*TaCDPK7D*	4D	482773327	482777407	1648	548	2
TraesCS5A02G463100	*TaCDPK8A*	5A	643328443	643333346	1606	534	2
TraesCS5B02G474500	*TaCDPK8B*	5B	647940529	647944797	1609	535	1
TraesCS5D02G475900	*TaCDPK8D*	5D	515114772	515119147	1606	534	2
TraesCS4A02G283400	*TaCDPK9A*	4A	590277244	590280756	1711	569	1
TraesCS4B02G030100	*TaCDPK9B*	4B	22272751	22276844	1804	600	2
TraesCS4D02G027600	*TaCDPK9D*	4D	12408039	12412107	1714	570	1
TraesCS5A02G426500	*TaCDPK10A*	5A	611564144	611567697	1885	627	1
TraesCS5B02G428400	*TaCDPK10B*	5B	604063931	604067708	1804	600	2
TraesCS5D02G434500	*TaCDPK10D*	5D	489904535	489908838	1876	624	2
TraesCS5A02G426800	*TaCDPK11A*	5A	611647144	611653336	1834	610	2
TraesCS5B02G428700	*TaCDPK11B*	5B	604158345	604164480	1762	586	1
TraesCS5D02G434800	*TaCDPK11D*	5D	490026551	490035692	1762	586	2
TraesCS2A02G407200	*TaCDPK12A*	2A	662905054	662907850	1621	539	1
TraesCS2B02G424900	*TaCDPK12B*	2B	610924597	610927456	1615	537	1
TraesCS2D02G404200	*TaCDPK12D*	2D	518612930	518615894	1606	534	1
TraesCS2A02G456100	*TaCDPK13A*	2A	704772804	704777045	1678	558	1
TraesCS2B02G478100	*TaCDPK13B*	2B	675352156	675356480	1681	559	1
TraesCS2D02G456400	*TaCDPK13D*	2D	564091471	564095952	1672	556	1

TraesCS1A02G309400	*TaCDPK14A*	1A	500341220	500343586	1501	499	2
TraesCS1B02G320400	*TaCDPK14B*	1B	544724734	544727121	1555	517	1
TraesCS1D02G308800	*TaCDPK14D*	1D	404968654	404971054	1555	517	2
TraesCS1A02G424200	*TaCDPK15A*	1A	579296187	579299659	1384	460	1
TraesCS1B02G456700	*TaCDPK15B*	1B	671199121	671202722	1384	460	1
TraesCS1D02G433400	*TaCDPK15D*	1D	482836356	482839907	1384	460	1
TraesCS1A02G292200	*TaCDPK16A*	1A	487735997	487741826	1657	551	2
TraesCS1B02G301600	*TaCDPK16B*	1B	522055416	522061604	1651	549	1
TraesCS1D02G290700	*TaCDPK16D*	1D	388645449	388653819	1651	549	1
TraesCS2A02G248200	*TaCDPK17A*	2A	369498016	369503089	1660	552	3
TraesCS2B02G269200	*TaCDPK17B*	2B	364729261	364734398	1660	552	3
TraesCS2D02G249900	*TaCDPK17D*	2D	296848612	296853319	1648	548	2
TraesCS2A02G239900	*TaCDPK18A*	2A	333724200	333771117	1531	509	1
TraesCS2D02G245700	*TaCDPK18D*	2D	284512874	284577109	1531	509	1
TraesCS2A02G223100	*TaCDPK19A*	2A	215747549	215752196	1579	525	3
TraesCS2B02G248700	*TaCDPK19B*	2B	255050988	255055240	1489	495	3
TraesCS2D02G229100	*TaCDPK19D*	2D	198459343	198463234	1600	532	3
TraesCS2A02G199700	*TaCDPK20A*	2A	172214508	172218294	1633	543	1
TraesCS2B02G227000	*TaCDPK20B*	2B	217789831	217793781	1633	543	1
TraesCS2D02G207400	*TaCDPK20D*	2D	161373419	161377378	1633	543	1
TraesCS7A02G267000	*TaCDPK21A*	7A	270041942	270044241	1753	583	1
TraesCS7B02G165200	*TaCDPK21B*	7B	228362103	228364342	1735	577	1
TraesCS7D02G267700	*TaCDPK21D*	7D	251373395	251375719	1756	584	1
TraesCS5A02G298500	*TaCDPK22A*	5A	505609485	505613419	1648	548	1
TraesCS5B02G297700	*TaCDPK22B*	5B	480291631	480295050	1660	552	1
TraesCS5D02G305200	*TaCDPK22D*	5D	400278000	400281717	1660	552	1
TraesCS4A02G206200	*TaCDPK24A*	4A	499341057	499345357	1543	513	2
TraesCS4B02G109800	*TaCDPK24B*	4B	122519855	122524340	1543	513	2
TraesCS4D02G107200	*TaCDPK24D*	4D	85578813	85582953	1546	514	1
TraesCS4A02G187900	*TaCDPK25A*	4A	466925867	466928439	1600	532	1
TraesCS4B02G130300	*TaCDPK25B*	4B	171025852	171028751	1600	532	1
TraesCS4D02G125500	*TaCDPK25D*	4D	109909795	109912155	1600	532	1
TraesCS5A02G138300	*TaCDPK26A*	5A	310872226	310875589	1627	541	1
TraesCS5B02G137600	*TaCDPK26B*	5B	259538033	259540793	1621	539	1
TraesCS5D02G153800	*TaCDPK26D*	5D	242356551	242359441	1627	541	1
TraesCS5B02G109300	*TaCDPK27B*	5B	153468195	153504183	1876	624	1
TraesCS5D02G124000	*TaCDPK27D*	5D	184872741	184896103	1876	624	1
TraesCS5A02G107900	*TaCDPK28A*	5A	191035649	191040409	1696	564	4
TraesCS5B02G115400	*TaCDPK28B*	5B	192379196	192383950	1591	529	2
TraesCS5D02G122800	*TaCDPK28D*	5D	177680127	177684898	1591	529	4
TraesCS5A02G118200	*TaCDPK29A*	5A	242531195	242538426	1663	553	1
TraesCS5B02G112900	*TaCDPK29B*	5B	177640656	177647588	1654	550	2
TraesCS5D02G119100	*TaCDPK29D*	5D	161449229	161460176	1675	557	2

Table S2

Wheat and rice CDPK orthologs based on phylogenetic analysis

Gene ID	Homolog in Rice	% identity
TaCDPK1A	*OsCDPK1*	89.38
TaCDPK1B	*OsCDPK1*	89.38
TaCDPK1D	*OsCDPK1*	89.38
TaCDPK2A	*OsCDPK2*	91.31
TaCDPK2B	*OsCDPK2*	90.54
TaCDPK2D	*OsCDPK2*	91.31
TaCDPK3A	*OsCDPK3*	93.28
TaCDPK3B	*OsCDPK3*	91.11
TaCDPK3D	*OsCDPK3*	92.01
TaCDPK4A	*OsCDPK4*	87.36
TaCDPK4B	*OsCDPK4*	87.36
TaCDPK4UN	*OsCDPK4*	87.36
TaCDPK5A	*OsCDPK5*	89.45
TaCDPK5B	*OsCDPK5*	90
TaCDPK5D	*OsCDPK5*	88.34
TaCDPK7A	*OsCDPK7*	80.22
TaCDPK7B	*OsCDPK7*	81.45
TaCDPK7D	*OsCDPK7*	81.09
TaCDPK8A	*OsCDPK8*	84.23
TaCDPK8B	*OsCDPK8*	84.04
TaCDPK8D	*OsCDPK8*	83.86
TaCDPK9A	*OsCDPK9*	78.26
TaCDPK9B	*OsCDPK9*	73.76
TaCDPK10A	*OsCDPK10*	80.7
TaCDPK10B	*OsCDPK10*	79.52
TaCDPK10D	*OsCDPK10*	79.49
TaCDPK11A	*OsCDPK11*	79.67
TaCDPK11B	*OsCDPK11*	83.11
TaCDPK11D	*OsCDPK11*	83.28
TaCDPK12A	*OsCDPK12*	71.16
TaCDPK12B	*OsCDPK12*	80.89
TaCDPK12D	*OsCDPK12*	81.34
TaCDPK13A	*OsCDPK13*	89.96
TaCDPK13B	*OsCDPK13*	91.06
TaCDPK13D	*OsCDPK13*	90.47
TaCDPK14A	*OsCDPK14*	75.67
TaCDPK14B	*OsCDPK14*	86.23
TaCDPK14D	*OsCDPK14*	84.09

TaCDPK15A	*OsCDPK15*	79.7
TaCDPK15B	*OsCDPK15*	79.15
TaCDPK15D	*OsCDPK15*	79.7
TaCDPK16A	*OsCDPK16*	93.85
TaCDPK16B	*OsCDPK16*	92.56
TaCDPK16D	*OsCDPK16*	94.37
TaCDPK17A	*OsCDPK17*	81.99
TaCDPK17B	*OsCDPK17*	81.82
TaCDPK17D	*OsCDPK17*	84.15
TaCDPK18A	*OsCDPK18*	90.23
TaCDPK18D	*OsCDPK18*	90.04
TaCDPK19A	*OsCDPK19*	92.12
TaCDPK19B	*OsCDPK19*	83.86
TaCDPK19D	*OsCDPK19*	93.25
TaCDPK20A	*OsCDPK20*	88.41
TaCDPK20B	*OsCDPK20*	88.95
TaCDPK20D	*OsCDPK20*	88.29
TaCDPK21A	*OsCDPK21*	74.37
TaCDPK21B	*OsCDPK21*	75.47
TaCDPK21D	*OsCDPK21*	73.15
TaCDPK22A	*OsCDPK22*	68.51
TaCDPK22B	*OsCDPK22*	67.3
TaCDPK22D	*OsCDPK22*	67.01
TaCDPK24A	*OsCDPK24*	91.62
TaCDPK24B	*OsCDPK24*	91.62
TaCDPK24D	*OsCDPK24*	92.41
TaCDPK25A	*OsCDPK25*	79.48
TaCDPK25B	*OsCDPK25*	78.56
TaCDPK25D	*OsCDPK25*	79.3
TaCDPK26A	*OsCDPK26*	77.6
TaCDPK26B	*OsCDPK26*	77.45
TaCDPK26D	*OsCDPK26*	77.31
TaCDPK27B	*OsCDPK27*	80.29
TaCDPK27D	*OsCDPK27*	80.45
TaCDPK28A	*OsCDPK28*	88.24
TaCDPK28B	*OsCDPK28*	86.57
TaCDPK28D	*OsCDPK28*	87.13
TaCDPK29A	*OsCDPK29*	79.47
TaCDPK29B	*OsCDPK29*	78.38
TaCDPK29D	*OsCDPK29*	79.61

Table S3
Primers Used

Primer name	Primer 5′-3′	Purpose
TaCDPK7-V1-S	5'- TAGCTAGCTGATTAATTAAATGGGCAATCAGAATGGGAC -3'	VIGS
TaCDPK7-V1-AS	5'- TTGCTAGCTGAGCGGCCGCAAGCTTCCGGCCAAGGATAT -3'	VIGS
TaCDPK7-V2-S	5'- TAGCTAGCTGATTAATTAACCTTCAGAGCGTTTGAAAGC -3'	VIGS
TaCDPK7-V2-AS	5'- TTGCTAGCTGAGCGGCCGCCAAAAGTGATTACGCCTCTA -3'	VIGS
QF-TaCDPK3	5'- GCCGCCGCCAGGAAGAATAAG -3'	qRT-PCR
QR-TaCDPK3	5'- CAGCAGGTACTTGTCGTCGATCC -3'	qRT-PCR
QF-TaCDPK5	5'- GTGATCTTATGGAAGCGGCGGATG -3'	qRT-PCR
QR-TaCDPK5	5'- GGCTGCCACAAGATGTTCCTCTC -3'	qRT-PCR
QF-TaCDPK7	5'- TGCTGATGGGATTGATGAGGAACG -3'	qRT-PCR
QR-TaCDPK7	5'- GCGGTGGGCTTGAACGAGTC -3'	qRT-PCR
QF-TaCDPK11	5'- AGGGTGATCGTCGGCGTCATC -3'	qRT-PCR
QR-TaCDPK11	5'- GCGGCGAGTCCTCCTGATTATTG -3'	qRT-PCR
QF-TaCDPK22	5'- GACGGTACTTTGACGCTTGAGGAG-3'	qRT-PCR
QR-TaCDPK22	5'- AGTGTCCAAGGTGCCATTTCCATC -3'	qRT-PCR
QF-TaCDPK25	5'- GAGGCTGACACTGACAATGATGGG-3'	qRT-PCR
QR-TaCDPK25	5'- GGTTCGTTGGCTCGGTGCTG -3'	qRT-PCR
QF-TaCDPK26	5'- GAGGCTGACACTGACAATGATGGG -3'	qRT-PCR
QR-TaCDPK26	5'- GGTTCGTTGGCTCGGTGCTG -3'	qRT-PCR
QF-TaCDPK27	5'- GGATATTCGAGCAGGTGCTGAAGG -3'	qRT-PCR
QR-TaCDPK27	5'- ACGGGTGGCAGAGGACTTGG -3'	qRT-PCR
QF-TaEF	5'- TGGTGTCATCAAGCCTGGTATGGT -3'	qRT-PCR
QR-TaEF	5'- ACTCATGGTGCATCTCAACGGACT -3'	qRT-PCR
QF-TaPR1	5'- GAGAATGCAGACGCCCAAGC -3'	qRT-PCR
QR-TaPR1	5'- CTGGAGCTTGCAGTCGTTGATC -3'	qRT-PCR
QF-TaPR2	5'- AGGATGTTGCTTCCATGTTTGCCG -3'	qRT-PCR
QR-TaPR2	5'- AAGTAGATGCGCATGCCGTTGATG -3'	qRT-PCR
QF-TaPR5	5'- CAAGCAGTGGTATCAACGCAGAG -3'	qRT-PCR
QR-TaPR5	5'- GTGAAGCCACAGTTGTTCTTGATGTT -3'	qRT-PCR
QF-TaCAT1	5'- TGCCTGTGTTTTTTATCCGAGA -3'	qRT-PCR
QR-TaCAT1	5'- CTGCTGATTAAGGTGTAGGTGTTGA -3'	qRT-PCR

Table S4
TaCDPKs Physio-chemical properties

Name	GRAVY	Aliphatic Index	Predicted localization	Instability Index	MW (k/Da)	pI
TaCDPK1A	-0.478	79.86	mito, chlo	43.2	58.4	5.79
TaCDPK1B	-0.478	79.86	mito, chlo	43.2	58.37	5.79
TaCDPK1D	-0.474	80.06	mito, chlo	43.57	58.37	5.79
TaCDPK2A	-0.403	80.08	chlo, cyto, plas, vacu, nucl	38.46	57.05	5.51
TaCDPK2B	-0.414	79.71	chlo, cyto, plas, nucl, vacu	37.56	57.22	5.46
TaCDPK2D	-0.41	79.52	chlo, cyto, plas, vacu, nucl	37.57	57.15	5.51
TaCDPK3A	-0.39	84.17	chlo, cyto, E.R., mito, pero, cysk	42.8	60.96	6.38
TaCDPK3B	-0.408	84.94	cyto, chlo, cyto_nucl, E.R., mito, pero	41.14	60.74	6.5
TaCDPK3D	-0.419	83.7	cyto, chlo, E.R., mito, pero, cysk	41.82	60.82	6.38
TaCDPK4A	-0.456	83.62	cyto, nucl, mito, plas	43.48	57.63	8.35
TaCDPK4B	-0.456	83.62	cyto, nucl, mito, plas	43.48	57.63	8.35
TaCDPK4UN	-0.451	83.65	cyto, nucl, mito, plas	43.41	57.7	8.35
TaCDPK5A	-0.257	83.58	mito, chlo_mito, chlo, cyto	42.92	60.65	5.3
TaCDPK5B	-0.254	83.7	mito, chlo_mito, chlo	42.88	60.45	5.39
TaCDPK5D	-0.271	83.31	mito, chlo_mito, chlo	42.45	59.63	5.39
TaCDPK7A	-0.241	92.82	cyto, mito, vacu	34.84	58.27	5.43
TaCDPK7B	-0.294	90.2	cyto, nucl, mito, pero	32.94	61.33	5.23
TaCDPK7D	-0.304	90.22	cyto, nucl, mito, vacu	34.62	61.32	5.39
TaCDPK8A	-0.448	81.82	mito, chlo, cyto, nucl	40.73	59.83	6.43

TaCDPK8B	-0.464	80.93	E.R., cyto, pero, cyto_nucl, chlo, mito	40.89	59.93	6.31
TaCDPK8D	-0.45	81.46	mito, cyto, chlo, nucl	41.61	59.81	6.43
TaCDPK9A	-0.403	80.98	chlo, extr, vacu, nucl	38.1	63.18	8.67
TaCDPK9B	-0.372	83.3	chlo, vacu, extr, nucl	40.68	66.8	8.82
TaCDPK9D	-0.375	82.4	chlo, extr, vacu, nucl	37.96	63.21	8.89
TaCDPK10A	-0.311	83.24	chlo, vacu, mito, pero	39.13	68.45	6.04
TaCDPK10B	-0.312	82.1	chlo, vacu, mito, pero	39.51	65.72	5.99
TaCDPK10D	-0.303	83.64	chlo, vacu, mito, pero	39.45	68.22	6.04
TaCDPK11A	-0.289	85.05	cyto, chlo, plas, nucl, mito, pero	45.29	67.09	5.45
TaCDPK11B	-0.331	81.91	chlo, cyto, nucl, mito, plas, E.R., pero	42.03	64.2	5.37
TaCDPK11D	-0.334	82.24	cyto, chlo, nucl, mito, plas, pero	42.36	64.26	5.38
TaCDPK12A	-0.326	81.69	cyto, nucl, chlo, pero	47.18	53.21	8.49
TaCDPK12B	-0.432	80.09	cyto, nucl, chlo	46.06	60.06	6.28
TaCDPK12D	-0.419	79.27	cyto, nucl, pero	45.4	59.61	6.09
TaCDPK13A	-0.324	83.82	mito, chlo_mito, chlo	40.47	61.48	5.49
TaCDPK13B	-0.317	83.85	mito, chlo_mito, chlo	39.99	61.47	5.43
TaCDPK13D	-0.347	83.4	mito, chlo_mito, chlo	41.02	61.39	5.43
TaCDPK14A	-0.246	85.83	cyto, nucl, mito, E.R.	29.13	55.99	5.97
TaCDPK14B	-0.395	79.26	chlo, mito, cyto	34.52	57.41	6.59
TaCDPK14D	-0.383	79.07	mito, pero, chlo, vacu	34	57.32	6.24
TaCDPK15A	-0.394	84.83	cysk, cyto, nucl	38.83	52.19	5.5
TaCDPK15B	-0.404	84.61	cysk, cyto, nucl	40.27	52.17	5.54
TaCDPK15D	-0.399	84.61	cysk, cyto, nucl	39.43	52.21	5.5

TaCDPK16A	-0.389	82.67	cyto, cyto_nucl, chlo, mito, E.R., pero, cysk	43.44	60.96	6.29
TaCDPK16B	-0.389	83.15	cyto, cyto_nucl, chlo, mito, E.R., pero, cysk	41.91	60.9	6.2
TaCDPK16D	-0.389	83.15	cyto, cyto_nucl, chlo, mito, E.R., pero, cysk	42.29	60.89	6.2
TaCDPK17A	-0.258	86.41	chlo, vacu, nucl, plas, extr	40.66	60.34	5.2
TaCDPK17B	-0.26	86.59	chlo, vacu, E.R., nucl, plas	40.93	60.35	5.2
TaCDPK17D	-0.259	87.04	vacu, chlo, nucl, mito, plas, extr, E.R.	40.8	60.03	5.2
TaCDPK18A	-0.419	83.73	chlo, cyto, plas	34.97	57.03	7.21
TaCDPK18D	-0.431	82.57	chlo, cyto, nucl	35.51	57.02	7.66
TaCDPK19A	-0.405	78.21	cyto, cyto_nucl, chlo, nucl, vacu	41.03	58.65	5.55
TaCDPK19B	-0.487	75.58	chlo, cyto, nucl	40.58	53.64	5.61
TaCDPK19D	-0.401	78.83	chlo, cyto, nucl	41.23	59.37	5.63
TaCDPK20A	-0.452	82.76	E.R., cyto, nucl, extr, chlo, vacu	36.67	60.96	6.59
TaCDPK20B	-0.461	82.23	E.R., cyto, nucl, extr, chlo, vacu	37.63	60.95	6.59
TaCDPK20D	-0.451	82.95	E.R., cyto, nucl, extr, chlo, vacu	38.14	60.96	6.59
TaCDPK21A	-0.438	76.9	chlo, cyto, cyto_nucl, mito, nucl, E.R.	41.65	64.08	8.44
TaCDPK21B	-0.442	76.85	chlo, cyto, cyto_nucl, mito, nucl, E.R.	40.86	63.53	8.44
TaCDPK21D	-0.439	76.27	chlo, cyto, cyto_nucl, mito, nucl, E.R.	40.82	64.23	8.26
TaCDPK22A	-0.457	78.54	chlo, mito, cyto_mito, cyto	51.33	61.22	5.46
TaCDPK22B	-0.467	79.75	chlo, mito, cyto, plas	50.17	61.78	5.4
TaCDPK22D	-0.46	78.9	mito, chlo, cyto_mito, cyto, plas	51.01	61.87	5.3

TaCDPK24A	-0.27	84.27	chlo, E.R.	38.24	56.7	5.4
TaCDPK24B	-0.274	84.07	chlo, E.R.	39.22	56.7	5.3
TaCDPK24D	-0.266	84.47	chlo, E.R., mito	38.59	56.81	5.4
TaCDPK25A	-0.413	80.36	cyto, pero, chlo, nucl, mito	37.67	58.77	5.61
TaCDPK25B	-0.405	81.09	cyto, mito, pero, chlo	37.65	58.78	5.81
TaCDPK25D	-0.425	79.81	cyto, pero, chlo, nucl	37.73	58.77	5.82
TaCDPK26A	-0.455	75.62	cyto, pero, chlo, nucl, mito	34.84	59.61	5.02
TaCDPK26B	-0.433	75.9	cyto, mito, pero, chlo	35.16	59.4	4.92
TaCDPK26D	-0.46	75.27	cyto, pero, chlo, nucl	35.94	59.69	4.99
TaCDPK27B	-0.433	78	chlo, nucl, cyto, mito, plas	55.38	68.9	5.6
TaCDPK27D	-0.454	77.37	chlo, nucl, cyto, mito, plas	54.88	69	5.54
TaCDPK28A	0.285	85.52	chlo, cyto, plas, nucl, cysk_nucl, vacu	36.63	57.32	5.42
TaCDPK28B	-0.249	86.28	chlo, cyto, plas, nucl, cysk_nucl, vacu	38.01	58.29	5.47
TaCDPK28D	-0.248	86.28	chlo, cyto, plas, nucl, cysk_nucl, vacu	38.17	58.26	5.47
TaCDPK29A	-0.344	85.68	chlo, mito, E.R.	46.44	62.1	6.37
TaCDPK29B	-0.327	86.15	mito, chlo, cyto, plas	46.91	61.58	5.95
TaCDPK29D	-0.332	85.58	mito, chlo, cyto, plas	48.62	62.48	5.91

Table S5

Tandem duplication events in wheat *CDPK* genes

Cluster number	Chromosome	Gene	Start Site	End Site
1	5	*TaCDPK10A*	611564144	611567697
	5	*TaCDPK11A*	611647144	611653336
2	5	*TaCDPK10B*	604063931	604067708
	5	*TaCDPK11B*	604158345	604164480
3	5	*TaCDPK26D*	242356551	242359441
	5	*TaCDPK29A*	242531195	242538426
4	5	*TaCDPK28D*	177680127	177684898
	5	*TaCDPK29B*	177640656	177647588
